# Reassessing the role of climate change in the Tupi expansion (South America, 5000–500 BP)

**DOI:** 10.1098/rsif.2021.0499

**Published:** 2021-10-06

**Authors:** Jonas Gregorio de Souza, Francisco Silva Noelli, Marco Madella

**Affiliations:** ^1^ Department of Humanities, Culture and Socio-Ecological Dynamics group (CaSEs), Universitat Pompeu Fabra, Barcelona, Spain; ^2^ Centro de Arqueologia (UNIARQ), Faculdade de Letras, Universidade de Lisboa, Lisbon, Portugal; ^3^ Institució Catalana de Recerca i Estudis Avançats (ICREA), Barcelona, Spain; ^4^ School of Geography, Archaeology and Environmental Studies, The University of the Witwatersrand, Johannesburg, South Africa

**Keywords:** archaeology, South America, climate change, simulation, demic diffusion

## Abstract

The expansion of forest farmers across tropical lowland South America during the Late Holocene has long been connected to climate change. The more humid conditions established during the Late Holocene are assumed to have driven the expansion of forests, which would have facilitated the dispersal of cultures that practised agroforestry. The Tupi, a language family of widespread distribution in South America, occupies a central place in the debate. Not only are they one of the largest families in the continent, but their expansion from an Amazonian homeland has long been hypothesized to have followed forested environments wherever they settled. Here, we assess that hypothesis using a simulation approach. We employ equation-based and cellular automaton models, simulating demic-diffusion processes under two different scenarios: a null model in which all land cells can be equally settled, and an alternative model in which non-forested cells cannot be settled or delay the expansion. We show that including land cover as a constraint to movement results in a better approximation of the Tupi expansion as reconstructed by archaeology and linguistics.

## 1. Introduction

The Late Holocene in South America was a period of significant ecological and social transformation with the establishment of a modern climate and the expansion of cultures and languages. Starting *ca* 4000–3000 BP, wetter conditions drove the expansion of forests in regions influenced by the South American summer monsoon, particularly the southern Amazon and southeastern Brazil [1,2]. Parallel to climate change, the archaeological record and historical linguistics show the dispersal of traditions and language families associated with plant cultivation and forest management in the South American tropical lowlands [3–6].

The territorial extent of archaeological cultures related to the spread of ceramics and farming mirrors the distribution of the largest language families in South America, suggesting that culture and language spread was a consequence of population growth and expansion from centres of domestication [7–10]. The concurrence of precipitation increase, forest expansion and population dispersal suggest that the establishment of modern climatic conditions and the geographical extent of current biomes favoured the expansion of forest agriculturists and their languages across the tropical lowlands of South America [3,11].

**Figure 1.**
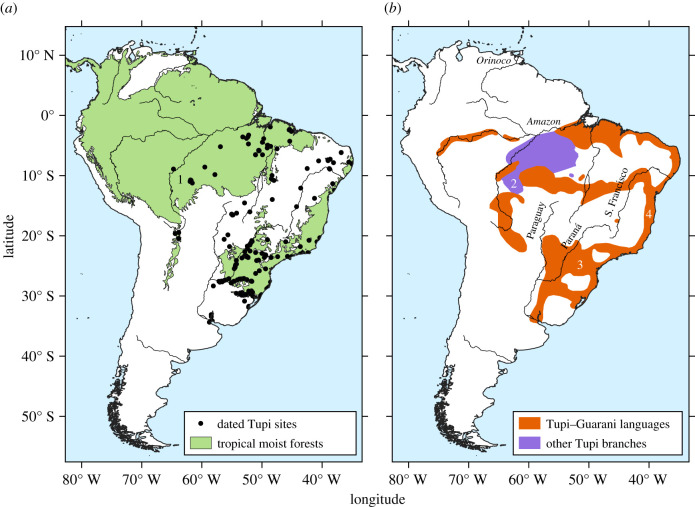
Distribution of Tupi sites and languages. (*a*) Archaeological sites with ^14^C dates [4] and tropical forest biome [12]. 1 = earliest dated site (Urupá, 5070 ± 60 BP) [13]. (*b*) Historical distribution of Tupi speakers [14–16]. 2 = probable homeland of the Tupi family; 3 = Guarani languages; 4 = Tupinambá languages.

Here, we employ computer simulations to assess the role of climate change in the expansion of one of those cultures, the Tupi, whose languages and material culture cover a vast territory (figure 1). The chronology and causes of the Tupi expansion have been one of the most debated questions of South American archaeology [8], and the coincidence between the distribution of Tupi sites and forests outside of the Amazon suggests that Late Holocene shifts in forest-savannah borders may have influenced the Tupi dispersal, making them the ideal case for testing the role of climate change in human migrations [11,17].

### 1.1. The Tupi expansion

The Tupi (or Tupían) language family comprises over 40 languages, divided into 10 subgroups [18–20]. The highest genetic diversity within the family is found in the southwestern Amazon, with half of the subgroups restricted to the Brazilian state of Rondônia, the most probable Tupi homeland [21].

Among the subgroups of the Tupi family, Tupi–Guarani is the most widespread. Speakers of Tupi–Guarani languages occupied most of the Brazilian coast and hinterland of the Paraná Basin (figure 1) [22–25]. The first archaeological models for the Tupi–Guarani dispersal assumed an initial split in the central Amazon, with parallel expansion routes along the Atlantic coast and the Paraná Basin, corresponding to the two branches that effectively left the Amazon: the Tupinambá and Guarani, respectively [7,8]. Later archaeological models shifted the hypothetical Tupi–Guarani homeland to the southwestern Amazon, based on the linguistic evidence for the Tupi family as a whole [16]. The Tupi–Guarani languages, however, have their highest diversity in the eastern Amazon [19,23,24,26]. Archaeological models that incorporate this information suggest a southward migration of the Guarani along the Paraná basin headwaters, near central Brazil, or a return of that branch to the southwestern Amazon before its southward displacement [15,27].

According to glottochronological estimates, the Tupi started to diverge *ca* 5000 BP [18,28,29]. The Tupi–Guarani subgroup is much shallower, with estimates of its initial split varying between *ca* 2500 and 1000 BP [22,28–30]. The earlier boundary (*ca* 2500 BP) agrees with the archaeological chronology, but the percentage of shared cognates between Tupi–Guarani languages appears to support a more recent separation [25,30]. Whatever the case, it is clear that there was a period of stasis in the Amazon, during which the several Tupi subgroups were established, followed by the continental expansion of one of those subgroups, the Tupi–Guarani—an event that has been described as an ‘explosion’ [30].

### 1.2. Archaeological correlates

The Tupi expansion has its material correlate in the appearance of polychrome and corrugated pottery outside of the Amazon. This material culture package was conventionally called ‘Tupiguarani Tradition’, in reference to the Tupi–Guarani languages [31]. Originally, two ceramic styles were recognized, corresponding to the Tupinambá (Atlantic coast) and Guarani (Paraná Basin) branches. More recently, however, other regional styles have been identified [15,32]. Because of the uncertainties in the regional subdivisions, and to avoid association with particular groups, we will refer to the whole archaeological complex as Tupi.

The southern part of the Tupi territory has the highest number of dates, attesting an arrival in the Paraná river at least 2010 ± 75 BP (2090–1740 cal BP; all dates calibrated with the ShCal20 curve [33] and reported using the 2*σ* interval; electronic supplementary material, figure S1) [34]. The Atlantic coast has yielded some unexpectedly early dates, as early as 2920 ± 70 BP (3220–2790 cal BP) [35]. A chronology after 1740 ± 90 BP (1825–1380 cal BP), however, is better supported for this region [35]. In the eastern Amazon, dates reaching 2430 ± 20 (2680–2340 cal BP) have been reported, confirming the deep chronology predicted by linguistics in this region. Such early dates are still seen with caution, however, and the majority of the sites in the eastern Amazon are later than 1670 ± 80 BP (1590–1350 cal BP) [36]. The earliest dates published so far reach back to 5070 ± 60 BP (5910–5600 cal BP) and come from the Brazilian state of Rondônia, the purported homeland of the Tupi family [13,37,38]. These dates have not been included in recent models of the Tupi chronology, which focused on the expansion of the Tupi–Guarani branch [4,39]. Nevertheless, they agree with the early chronology for other cultural developments in the southwestern Amazon, including plant cultivation [40], and are currently the only archaeological correlate for the initial divergence of the Tupi family.

The small number of dates for some regions confounds our understanding of the Tupi dispersal. Recently, space–time regressions employing different methods (bootstrapping and Monte Carlo simulations) were used to detect cultural dispersal while incorporating the uncertainty in the radiocarbon record; however, such applications did not find any significant correlation between distance from the origin (the earliest site(s) in the eastern Amazon) and arrival time [4,39]. Similarly, agent-based models failed to replicate the Tupi–Guarani chronology, at least when assuming an expansion starting in the eastern Amazon *ca* 2400 BP [4]. That does not mean that demic diffusion did not happen—only that the resolution of the available radiocarbon record is currently insufficient to detect its speed and direction [39]. Incorrect dates may also be skewing our understanding [4].

### 1.3. Relationship with climate change

The hypothesis that the Tupi expansion was caused by climate change is not new. Meggers [17] drew on the (now disproven) refuge theory to suggest that the expansion of the Tupi and other language families coincided with periods of aridity that forced the population to disperse across forest refuges. More recently, Iriarte *et al*. [11] relied on a summary of palaeoecological evidence to posit that forest expansion, not contraction, was the driver of the Tupi expansion. The increase in forested areas that could be exploited by the Tupi, it is argued, facilitated their dispersal from the southwestern Amazon to similar niches elsewhere [11]. Given the short distances separating the Amazon and the Atlantic forest, it is plausible that the advance (or retreat) of forests in the southern Amazon and southeastern Brazil would have created corridors (or barriers) that facilitated (or delayed) the Tupi dispersal.

The Tupi phenomenon needs to be understood as part of other cultural expansions in tropical lowland South America [4]. The expansions in question diffused the cultivation of domesticated plants and management of semi-domesticated species with different degrees of human impact on the forests—a package conventionally called polyculture agroforestry. The spread of this economic and material culture package has been related to increased precipitation and forest expansion at the Mid- to Late-Holocene transition [3]. The southwestern Amazon, where the Tupi homeland is located, was also the centre of origin of other language families of widespread distribution in tropical lowland South America, such as Arawak [41]. Furthermore, this region yielded some of the earliest evidence of forest management, plant domestication and adoption of exotic cultivars in the Amazon, reinforcing the association between language expansion and domestication centres [9,40,42–45].

Reliance on forest management by modern and historically recorded Tupi groups also speaks in favour of an environmental cause for their expansion [46]. Historically, the presence of an arboreal canopy was vital for the Guarani and Tupinambá in establishing their territories, as their economy was devised to function within the forest [47–49]. The ethnography of modern Tupi-speaking groups shows that settlement in a new area is preceded by the management of forests and the introduction of cultivated plant species [50]. There are abundant records of similar agroforestry practices among all Tupi populations [51–55]. Reconstructions of the proto-Tupi cultural vocabulary attest to the persistence of gardening and a range of cultivated plants from the origins of the language family to the present [6]. In summary, the hypothesis that Mid- to Late-Holocene forest expansion in South America was determinant in the dispersal of the Tupi deserves serious consideration.

To assess the relationship between climate change and the Tupi expansion, we employed computational modelling. Recent simulations of demographic expansions demonstrated that better approximations of reality are achieved when the environment is included as a constraint to movement [56–58]. However, the only agent-based simulations of demographic expansions in South America, including the Tupi, did not consider the influence of environmental change [4]. Recent attempts to correlate the Tupi chronology with changes in vegetation yielded promising results, but were restricted to the Paraná Basin and relied on visual inspection of the data [11].

We model demic-diffusion processes using commonly employed algorithms. Specifically, we applied an equation-based model (EBM) of front propagation with an underlying cost surface [56,57] and a cellular automaton (CA) simulating population growth and dispersal in a dynamic environment [4,59,60]. To test whether climate change had a role in the movement of the Tupi, we executed models in which all the tropical lowlands can be settled equally (which we refer to as the null hypothesis) and models in which settlement advances at a faster rate or is restricted to tropical moist forest environments (our alternative hypothesis). In the CA, forest extent is dynamically updated using climate-based vegetation models [61]. If forest advance and retraction influenced the Tupi expansion, it is expected that the inclusion of vegetation as a constraint for settlement should result in a better approximation of the observed chronology.

## 2. Material and methods

### 2.1. Chronology

For comparison with the simulated chronology, we used the Tupi subset (*n* = 392) of our previously published database of tropical lowland South American radiocarbon dates [4]. Dates were collected from published databases, articles, academic theses and reports [15,34,62] (see the electronic supplementary material, table S1 for the references). We excluded dates with questionable Tupi affiliation, dates with an unclear association between the date and the archaeological context, and dates that are not supported by the regional chronology or by other dates from the same site [4]. Because most dates in a region tend to be later than first arrival times, to avoid the bias that could be created towards models with later simulated arrival times, we selected the earliest dates in 100 km spatial bins [57]. The final dataset contains 74 dates (electronic supplementary material, table S1).

### 2.2. Environment

We used the modern extent of South American biomes [12] as a cost surface in the EBM, attributing different weights to the biomes. For the environmental background in CA, there was the need to ideally use a reconstruction of land cover for South America over the last 6000 years. Biome reconstructions for South America based on fossil pollen records have focused on specific time slices (the Mid-Holocene, the last glacial maximum) and/or do not consist of continuous interpolated surfaces that can be directly incorporated in our model [2,63]. In Europe, good results have been obtained using landscape reconstruction algorithm (REVEALS and LOVE) [64–66], but those are of difficult application in lowland South America owing to the paucity of data for pollen productivity and rate of deposition. In addition, landscape reconstruction algorithms are based on assumptions that may be violated in the Neotropics [2].

For those reasons, palaeovegetation reconstructions based on general circulation models are currently the best alternative for our purpose [61,67,68]. We used the biome reconstructions of Costa *et al*. [61], which, among the available models, are the ones with the highest spatial and temporal resolution. They are based on a random forest classification algorithm trained on current vegetation and climate and fitted to palaeoclimate simulations from the Hadley Centre Coupled Model (HadCM3) at 1000-year intervals [61]. The model classifies South America into seven biomes based on plant functional groups and climate, broadly coinciding with accepted classifications [12].

### 2.3. Simulation

#### 2.3.1. Equation-based model

To provide a first estimate of arrival times, we followed Silva & Steele [56] and Russel *et al*. [57], including anisotropy in the EBM of the dispersal. By including a cost surface, the speed of the expansion is accelerated or slowed down. We estimated the speed of the propagation wave from two parameters: growth rate and mean displacement. The time-delayed adjustment of Fisher's reaction–diffusion equation [69], which considers the effect of generation lag in migration [70], was used to calculate the front speed as

2.1 $$\frac{2\sqrt{aD}}{1+a(\tau /2)},$$

where *a* is the initial growth rate, *D* is the diffusion coefficient and *τ* is the generation time. The diffusion coefficient is given by $\langle \Delta^{2}\rangle /4T$ [71], where ⟨Δ⟩ is the mean individual displacement per generation.

Given a propagation speed, we modelled arrival times based on the distance of each cell from the centre of origin. We calculated the least-cost distances using Dijkstra's algorithm [72] and the knight's move as implemented in R with the package *gdistance* [73]. Distances were calculated under two conditions: a null model in which movement occurs at the same rate in all land cells and an alternative model in which non-forested cells receive an extra cost, delaying the expansion in those biomes. Because we aimed to ascertain whether forested environments facilitated the Tupi dispersal, we only considered land cover for the friction surface. Future experiments should explore whether the inclusion of features such as rivers and terrain improves the accuracy of the model [56,57].

#### 2.3.2. Cellular automaton

To specifically test the role of a dynamic Late-Holocene environment in the Tupi expansion, we designed a simulation based on the architecture of previously published models of demic expansion in Eurasia and South America [4,59,60]. The model was implemented in Python, and the code (together with the R code for the EBM) is available in a public Zenodo repository (https://doi.org/10.5281/zenodo.4964642).

The simulation runs in discrete time and space, the latter consisting of a grid of 50 × 50 km cells covering South America in Albers Equal Area Projection. The cells' state consists of the current population, land cover category and arrival time. Each time step corresponds to a generation, during which the environment may be updated and, for every cell that is inhabited, population growth and dispersal methods are applied. The simulated date is recorded in a cell when it is converted to an inhabited cell for the first time.

Population growth and emigration are modelled as density-dependent processes, regulated by a theoretical maximum population density, which, for convenience, it is set to be equivalent to the carrying capacity (*K*) (see discussion in Read & LeBlanc [74]). The speed of demic diffusion is determined by the maximum growth rate, maximum emigration rate and generation time. We ran simulations in which all tropical biomes in the model of Costa *et al*. [61] could be settled, and others in which settlement was restricted to cells with tropical moist forest.

*Population growth:* for population growth, the logistic model is applied [75–77]. The population of a cell after growth at a time step (*N_t_*) is given by

2.2 $$N_{t}=\frac{KN_{0}}{(K-N_{0})e^{-a\tau }+N_{0}},$$

where *N*_0_ is the previous population, *τ* is the generation time and *a* is the intrinsic growth rate.

*Population dispersal:* models of density-dependent dispersal commonly assume a population density threshold past which the probability of emigration increases according to a function [78]. The marginal value theorem has been used to justify the pressure to emigrate owing to diminishing returns as the population increases [78]. Here, the rate of emigration at a given time step (*ɛ_t_*) is given by the equation of Best *et al*. [79] and Altwegg *et al*. [80]:

2.3 $$\varepsilon_{t}=\varepsilon {\left(\frac{N_{t}}{K}\right)}^{\gamma },$$

where *ɛ* is the emigration rate when the population is at *K*, and *γ* controls the shape of the dependence. We fix *γ* = 1, which results in the emigration rate increasing linearly with density.

Migrants are equally redistributed to the nearest eight cells. This resettlement distance (*ca* 50 km) is observed among Tupi populations and other groups of Amazonian farmers [47,50,81]. Cells that are densely settled are avoided, as are environmentally unsuitable cells. In those cases, the migrant population is redistributed only among the available, suitable cells. When emigration is not possible owing to the saturation of the neighbourhood around a cell, the population will continue to grow, converging to *K*.

In models where settlement is restricted to forested cells, the inhabitable area becomes discontinuous. In those cases, cells within a larger radius may be searched, and the population is allowed to jump over unsuitable areas if habitable cells are found. This procedure, known as leapfrogging, is commonly adopted in models where settlement is driven by maximizing environmental suitability [4,60]. Such long-distance moves are attested among the Tupi and other Amazonian societies. We limited the distance of leapfrogging to 150 km based on the ethnohistorical literature of long-range migrations, logistic expeditions and war incursions among Tupi populations [82,83]. Leapfrogging is restricted to cells at the borders of forested environments and only occurs when dispersal to the nearest cells is impossible. This is in line with the hypothesis of Tupi migration in ‘regional *nuclei*’ directed towards forested environments, separated by low-density or uninhabited areas in open landscapes [15,34]. Leapfrogging is suggested to have been a rare event, as the preparation of forested environments and plant transportation would encourage slower movement towards adjacent territories [16].

Finally, if a settled cell becomes unsuitable after the environmental update, its population attempts to disperse to available, suitable cells in the neighbourhood, following the same rules stated above. If that is not possible, the population of the cell becomes extinct. We adopt this procedure because we are explicitly testing for the dependence of Tupi occupation on land cover—therefore, it is consistent that the population advances and retracts accompanying the forest dynamics.

### 2.4. Experiment and parametrization

In all experiments, expansion starts at the coordinates of the Tupi site with the earliest date [13]. The chosen site is within the boundaries of the hypothetical homeland of the Tupi language family [28]. We set the starting date to 5000 BP based on the estimates for the beginning of the Tupi expansion. This is later than the earliest reported radiocarbon date (5910–5600 cal BP). However, the earliest appearance of an archaeological culture does not necessarily correspond to the beginning of its expansion, as demonstrated, for example, by the delay of the Neolithic expansion in reaching Europe from the Near East [59].

To adjust the friction surface in the EBM, we kept the relative cost of traversing tropical forests equal to 1 (no extra cost) and changed the cost of other biomes. In the CA, every run starts with a single settled cell, whose population is initialized at *K*, immediately dispersing to the neighbouring cells. The simulation then runs for a number of generations until the approximate date of the European arrival, at *ca* 500 BP, is reached.

The following values were adopted for the model parameters (table 1).

— *Maximum population density:* a wide range of densities are observed among modern Amazonian populations, most of which are relatively low as a result of post-contact decline [50,84]. Archaeological estimates, by contrast, suggest higher densities in some areas [85,86]. We kept *K* constant at 1 person km^−2^. This relatively high density is supported by the ethnohistory of Tupi-speaking people, which abound with references to extended-family communal houses and villages of hundreds of dwellers [47,82,87,88].
— *Annual growth rate:* based on the ethnography of pre-industrial farmers and Neolithic skeletal data, growth rates varying from 2.4 to 4% have been reported in the literature, with the lower end of the interval being more common [56,60,89]. Among modern Amazonian populations, some of the fastest growth rates have been recorded, reaching *ca* 3–4%, probably owing to a recovery from recent epidemic-driven loss [90]. We tested values between 2 and 4% and chose a default value of 2.5%, which is close to the values adopted by previous simulations of the Neolithic expansion [59,60] and Neotropical farmer expansion [4].
— *Emigration rate:* the percentage of migrants per generation is difficult to derive from ethnographic data. We tested values between 10 and 50% and adopted the default value of 30% based on published dispersal kernels of pre-industrial farmers, and considering the percentage of the population that moves at least 50 km [91].

**Table 1.** Simulation parameters and values.

parameter	description	default value	swee**p value**s	unit
*K*	carrying capacity	1	constant	individuals km^−2^
*r*	annual growth rate	2.5	{2, 2.5, 3, 3.5, 4}	%
⟨Δ⟩	average displacement	50	{40, 45, 50, 55, 60}	km generation^−1^
***ɛ***	emigration rate at *K*	30	{20, 25, 30, 35, 40}	%
***τ***	generation time	30	constant	years

### 2.5. Evaluation

The uncertainties surrounding the Tupi chronology hinder the evaluation of simulated arrival times [4,39]. One method, common in demic-diffusion models, is to compare the simulated chronology with the earliest observed radiocarbon dates in different regions [4,57,60]. This is justified by the fact that most dates are expected to be considerably later than the first arrival time in a region. At the same time, this approach is sensitive to incorrect dates that may over or underestimate the first arrival times. While recognizing such problems, in the absence of a more reliable method and considering the limitations of the available Tupi chronology, we use the root mean square error (RMSE) between simulated arrival times at a given Tupi site and the earliest date at that site as a heuristic measure of model accuracy.

## 3. Results and discussion

**Figure 2.**
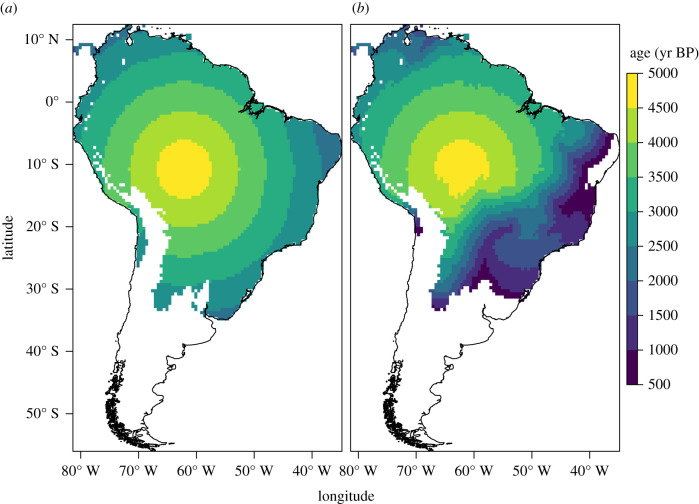
Simulated arrival times in the equation-based model using the default parameters. (*a*) Null hypothesis (cost of all biomes = 1). (*b*) Alternative hypothesis (cost of crossing biomes other than tropical moist forest = 4).

**Figure 3.**
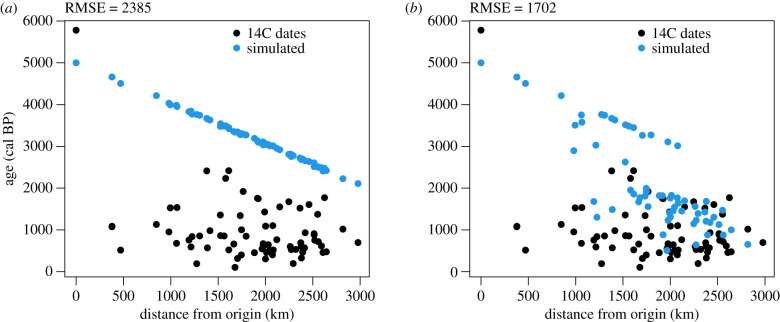
Plot of observed (median of calibrated dates) and simulated arrival times versus distances from the origin in the equation-based model using the default parameters. (*a*) Null hypothesis (cost of all biomes = 1). (*b*) Alternative hypothesis (cost of crossing biomes other than tropical moist forest = 4). RMSE = root mean square error between simulated and 14C dates.

**Figure 4.**
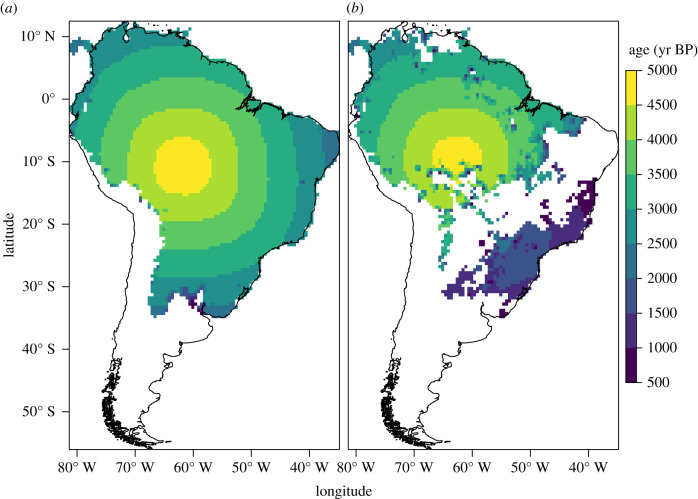
Simulated arrival times in the cellular automaton using the default parameters. (*a*) Null hypothesis (all land cells). (*b*) Alternative hypothesis (only tropical moist forests).

**Figure 5.**
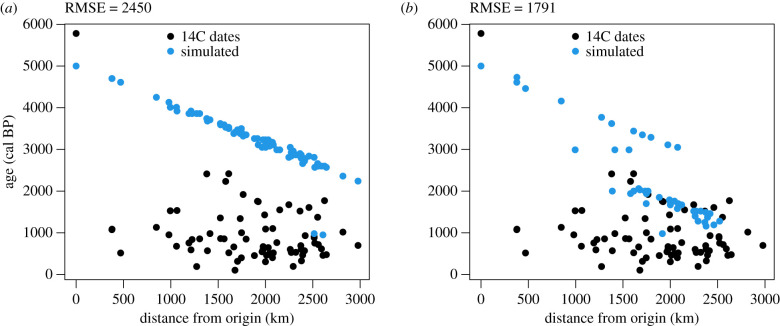
Plot of observed (median of calibrated dates) and simulated arrival times versus distances from the origin in the cellular automaton using the default parameters. (*a*) Null hypothesis (all land cells). (*b*) Alternative hypothesis (only tropical moist forests). RMSE = root mean square error between simulated and 14C dates.

**Figure 6.**
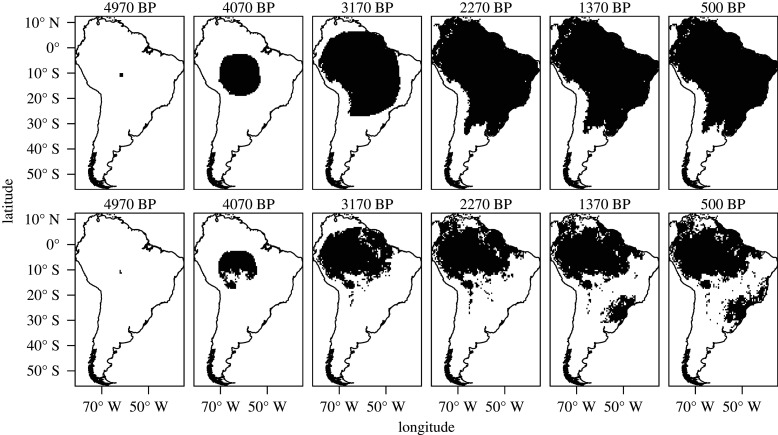
Time slices of the cellular automaton simulation showing occupied areas in 30-generation intervals up to 500 BP in the null model (top) and alternative model (bottom) using the default parameters.

The default parameter values in the null model result in a speed of advance of *ca* 1 km yr^−1^, consistent with the speed of demic-diffusion processes elsewhere [56,59,92]. Assuming the dispersal started *ca* 5000 BP, the null model greatly overestimates arrival times in the regions settled by the Tupi outside of the Amazon (figures 2–6). Setting the start of the expansion at 5800 BP (the earliest date published for a Tupi site) would result in even earlier simulated arrival times, and therefore a poorer fit.

In the EBM, moderately increasing the cost of crossing biomes other than tropical moist forests results in a better fit with the archaeological chronology (electronic supplementary material, table S2). Similarly, in the CA, restricting settlement to moist tropical forests creates a delay at *ca* 3000 BP and the second wave of advance after *ca* 2000 BP. As a result, simulated arrival times are closer to the observed radiocarbon dates in regions such as the Paraná Basin and the Atlantic coast (figures 2, 4 and 6; electronic supplementary material, table S3). This is coherent with the expectation that forest expansion between *ca* 3000 and 2000 BP, as inferred from the pollen records [1,2], opened corridors for the Tupi advance.

The expansion under the alternative hypothesis follows a pattern of ‘pulse and pause’. The second pulse of expansion is coherent with the model of an ‘explosion’ related to the spread of the Tupi–Guarani branch, following the long pause of the Tupi within the Amazon [30]. Similar dynamics have previously been suggested, at a smaller scale, for the Guarani occupation in the La Plata Basin [34]. A delay in the signal of the Neolithic demographic transition in the radiocarbon record of the La Plata Basin has been suggested to be because of the adaptation of a package of essentially tropical cultivars to the subtropics [93], which may have contributed to the lag of the Tupi in leaving the Amazon. As interesting as this hypothesis may be for future investigation, the current archaeological evidence suggests that the same Amazonian plants package was carried by the Tupi wherever they settled [34].

The alternative hypothesis offers a better qualitative approximation of the branching of the Tupi language family. The first wave of expansion is restricted to the Amazon, corresponding to the formation of the Tupi subgroups [18,19]. Once the corridors connecting the Amazon with the Atlantic forest are established, the second wave of expansion happens, mirroring the rapid diversification of the Tupi–Guarani branch. In summary, the simulation results support the hypothesis that forest expansion facilitated the dispersal of the Tupi [11].

However, the inclusion of vegetation also creates some disagreements with the archaeological and linguistic data. The most obvious weakness is observed in the regions furthest from the centre of origin, as in the case of the Paraná Delta or the northeastern Brazilian coast. If, in the null model, simulated arrival times are unacceptably early in those areas, in the alternative model they fail to be settled at all (figures 2, 4 and 6). Migration routes are also problematic. It is assumed that at least two migration routes have been followed by the Tupi as they left the Amazon: one towards the Paraná Basin, and a parallel one along the coast [8,16]. In the CA, however, only the purported migration from the southwestern Amazon to the Paraná Basin—and from there to most of the coast—is replicated (figures 4 and 6). Although this does correctly reproduce the Guarani dispersal according to some models, a route further to the east has been gaining more acceptance [15,16,27]. This preferred route is correctly reproduced in the EBM (figure 2). In both the EBM and the CA, however, the Tupinambá southward migration along the coast is absent, replaced by a movement in the opposite direction, which is now discredited [15]. In summary, although the broad pattern of the Tupi expansion seems to have been correctly captured, the precise migration routes as assumed by current linguistic and archaeological data have not been completely replicated. It is important to note, however, that a radial expansion of the Guarani and Tupinambá, the Tupi–Guarani branches that effectively left the Amazon, has been posited by some linguists and archaeologists [15,26]. Given our results, this possibility should be given further consideration.

It is possible that the Tupi dispersal was shaped by other factors besides forest expansion and social barriers could have been a key one. The Tupi expansion did not happen in a vacuum—and the presence of rival populations (either hunter–gatherers or farmers) has previously been hypothesized to have discouraged the settlement in some areas [34]. This argument could be made, for example, for the Amazon floodplain and the areas north of it, predominantly occupied by agricultural societies of the Saladoid–Barrancoid and similar complexes [94]. The absence of social barriers in our simulation resulted in the Tupi being free to settle the entire Amazon (figures 2, 4 and 6). We do not consider, however, that this scenario is valid for other areas outside the Amazon and within the Tupi expansion range. The avoidance of the *cerrado*, the central Brazilian savannah, for instance, cannot be attributed to rivalry with other groups, as societies inhabiting this biome are considered to have been less densely settled than the Tupi [95]. Similarly, at the Atlantic coast, the presence of large and sedentary maritime-based societies did not stop the Tupi wave of advance [96]. Therefore, we find that an environmental cause is still the best explanation for the pattern of Tupi settlement outside of the Amazon. Nevertheless, the inclusion of friction with other populations in future versions of the model may provide an evaluation of the social barrier hypothesis.

Another possibility to take into consideration is that not all the Tupi dispersal was a demographic phenomenon, but that some degree of cultural diffusion may have taken place, influencing the rate of spread. Adoption of language and material culture, rather than migration, has been suggested to explain other linguistic expansions in the Amazon, namely the Arawak [97]. However, cranial morphological data and genomic analysis of modern Tupi speakers confirm their common Amazonian ancestry and act as strong evidence that the Tupi expansion was mostly owing to demic diffusion [98,99].

A limitation that must be recognized in the current approach is the dependence on radiocarbon dates for evaluation. The number of Tupi dates is still relatively small when compared to applications in other parts of the world, and the chronology in some regions is still unresolved. The acquisition of new dates, or the exclusion of dates that may be found to be unreliable, would necessarily lead to a re-evaluation of our model results. One way to overcome this limitation would be to include other lines of evidence—for example, comparing the simulated routes of expansion with linguistic and cultural phylogenies [56,57]. Unfortunately, in our case, this is hindered by disagreements about the internal classification of the Tupi–Guarani languages and archaeological ceramic phases [25]. If those questions are resolved in the future, linguistic and material culture data can act as additional lines of evidence for the model evaluation.

Finally, we recognize that models are sensitive to initial conditions. Even in the null model, the fit with the archaeological data could be improved by using lower growth and emigration rates (electronic supplementary material, tables S2 and S3). A more serious problem is the dependence of the simulations on the quality of the underlying vegetation model. Although we selected the best palaeovegetation reconstruction available at the moment, at least for our purpose [61], we do not discard that in the future better results can be obtained by using an improved vegetation model.

## 4. Conclusion

The long tradition of archaeological and ethnographic research about the dispersal of the Tupi across the Neotropical lowlands has, recently, gained new interest thanks to the application of computational methods [4,39]. The hypothesis, postulated since at least the 1970s, that climate-driven forest expansion and retraction could have influenced human dispersals out of the Amazon has also been revived thanks to the accretion of palaeoenvironmental records [11]. Here, we reassess that hypothesis by employing a simulation approach. Assuming an origin of the Tupi language family in the southwestern Amazon at *ca* 5000 BP, we tested the null model of no environmental influence in their rate of expansion against an alternative model in which biomes other than tropical moist forests were more costly to traverse or were not settled.

Our results, albeit preliminary, bring further support to the role of Late-Holocene climate change in causing or facilitating the Tupi dispersal. We show that the inclusion of land cover as a factor that delays or restricts settlement results in better agreement with the archaeological chronology and with the reconstructed internal branching of the Tupi languages. However, some uncertainties remain, particularly concerning the specific migration routes and the controversies surrounding the Tupi radiocarbon record [4,39]. In the future, we expect that an increase in the number of dates with reliable contexts will lead to a re-evaluation of the models. We expect that the results of our model will motivate future hypothesis-driven research. Specifically, the suggested migration route towards the Atlantic coast should be re-evaluated given the simulation results by refining the chronology in the proposed migration corridors. Most importantly, interdisciplinary research combining the collection of new palaeovegetation records and radiocarbon dates in regions of Tupi occupation (particularly in the corridors connecting the Amazon and the Atlantic forest) would offer support to or contradict our simulation results. We also foresee that improved palaeovegetation models (either climate-based or pollen-based) will allow for more accurate simulations of the Tupi and other Amazonian expansions in the context of Late-Holocene climate change.

## Data accessibility

The data that support the findings of this study are included in this published article and its electronic supplementary material. The Python and R codes for running the simulations are available at https://doi.org/10.5281/zenodo.4964642.
